# Significant metabolic alterations in non-small cell lung cancer patients by epidermal growth factor receptor-targeted therapy and PD-1/PD-L1 immunotherapy

**DOI:** 10.3389/fphar.2022.949745

**Published:** 2022-08-11

**Authors:** Chen Yan, Dan Wu, Lingling Gan, Jun Wang, Wenyu Yang, Bei Xu

**Affiliations:** ^1^ Department of Clinical Pharmacy, Sichuan Cancer Hospital & Institute, Sichuan Cancer Center, School of Medicine, University of Electronic Science and Technology of China, Chengdu, China; ^2^ NHC Key Laboratory of Nuclear Technology Medical Transformation, Mianyang Central Hospital, School of Medicine, University of Electronic Science and Technology of China, Mianyang, China; ^3^ College of Medical Technology, Chengdu University of Traditional Chinese Medicine, Chengdu, China

**Keywords:** non-small cell lung cancer, epidermal growth factor receptor-targeted therapy, PD-1/PD-L1 immunotherapy, tumor metabolic reprogramming, untargeted metabolomics

## Abstract

**Background:** Cancer-related deaths are primarily attributable to lung cancer, of which non-small cell lung cancer (NSCLC) is the most common type. Molecular targeting therapy and antitumor immunotherapy have both made great strides in the treatment of NSCLC, but their underlying mechanisms remain unclear, especially from a metabolic perspective.

**Methods:** Herein, we used a nontargeted metabolomics approach based on liquid chromatography-mass spectrometry to analyze the metabolic response of NSCLC patients to epidermal growth factor receptor-tyrosine kinase inhibitors (EGFR-TKIs) or PD-1/PD-L1 inhibitors. Multiple analyses, including principal component analysis (PCA), orthogonal partial least squares-discriminant analysis (OPLS-DA) and pathway analysis, were used for metabolic data analysis. Additionally, differential metabolites were analysed and identified by publically available and integrated databases.

**Results:** After treatment with EGFR-TKIs or PD-1/PD-L1 inhibitors, glutamate/glutamine, phenylalanine, n-acetyl-l-leucine, n-acetyl-d-tryptophan, D-n-valine, arachidonic acid, and linoleic acid levels were significantly increased in patients with NSCLC, whereas carnitine, stearyl carnitine, palmitoyl carnitine, linoleic carnitine, and palmitic acid levels were markedly decreased. Compared with newly diagnosed, untreated patients, there were three shared metabolic pathways (phenylalanine metabolism, glycerophospholipid metabolism, and D-glutamine and D-glutamate metabolism) in the EGFR-TKIs or PD-1/PD-L1 inhibitor-treated groups, all of which were related to lipid and amino acid metabolism. Moreover, there were significant differences in lipid metabolism (glycerophospholipid metabolism and phosphatidylinositol signaling) and amino acid metabolism (tryptophan metabolism) between the EGFR-TKI and PD-1/PD-L1 inhibitor groups.

**Conclusion:** Our results show that EGFR-TKIs and PD-1/PD-L1 inhibitors induce changes in carnitine, amino acids, fatty acids, and lipids and alter related metabolic pathways in NSCLC patients. Endogenous metabolism changes occur due to drug action and might be indicative of antitumor therapeutic effect. These findings will provide new clues for identifying the antitumor mechanism of these two treatments from the perspective of metabolism.

## 1 Introduction

There were 19.29 million new cancer diagnoses in 2020, of which 2.2 million were lung cancers. Lung cancer accounts for 11.4% of the total cancer diagnoses, making it the second most common cancer worldwide (Sung et al., 2021). Additionally, 1.8 million people died of lung cancer in 2020, accounting for 18.0% of total cancer deaths (Sung et al., 2021). Lung cancer is the leading cause of cancer-related death. Among men, lung cancer is the most common cancer in 36 countries and the leading cause of cancer mortality in 93 countries (Sung et al., 2021). With global population growth, aging, and changing lifestyles, the burden of lung cancer is increasing.

Lung cancers are divided into two categories: non-small cell lung cancer (NSCLC) and small cell lung cancer. NSCLC is the most common type, accounting for approximately 80–85% of all lung cancer cases, and has the highest mortality rate (Singh et al., 2021). The current treatment of NSCLC is not only focused on surgical treatment, chemotherapy, radiotherapy, and comprehensive treatment but includes molecular targeted therapy and antitumor immunotherapy (Ghini et al., 2020; Wang et al., 2021). Epidermal growth factor receptor (EGFR) is the most common driver gene mutation of NSCLC, with approximately 35% of Asian patients and 60% of patients with lung adenocarcinomas (Pi et al., 2018; Yoon et al., 2020). EGFR tyrosine kinase inhibitors (EGFR-TKIs) are the most widely used targeted therapies and are remommended as first-line treatment for NSCLC patients with EGFR activating mutations (National Comprenhensive Cancer Network, 2017; He et al., 2021). These drugs improve progression-free survival and overall survival compared to conventional chemotherapy and are associated with fewer severe adverse events (Singh et al., 2021).

Antitumor immunotherapy is another effective and safe treatment for NSCLC. Immune checkpoint inhibitors are the most studied immunotherapies for NSCLC (Herzberg et al., 2017; Xiong et al., 2021). Immunotherapy differs from traditional chemotherapy and targeted therapy as it kills the tumor by overcoming immunosuppression and reactivating the patient’s own immune cells (Alexander et al., 2020). The immune checkpoint molecules PD-1 and its ligands, PD-L1 and PD-L2, are key therapeutic targets (Robert, 2020).

Tumorigenesis and development are closely related to metabolism (DeBerardinis and Chandel, 2016). Metabolic reprogramming is associated with tumorigenesis and is an important hallmark of cancer. Therefore, tumors are not only genetic diseases but also metabolic diseases (Hanahan and Weinberg, 2011). Mutations in tumor-related genes cause changes in multiple signaling pathways in cells, which reshapes the metabolism of tumor cells to enhance their survival and growth ability (Nagarajan et al., 2016). EGFR-TKIs and PD-1/PD-L1 inhibitors can inhibit tumor growth through a variety of signaling pathways, but evidence of their effect on metabolic pathways is limited. Therefore, exploring the effects of EGFR-TKIs and immunotherapy on metabolic pathways in NSCLC would be useful to elucidate the mechanism of these two new therapies and provide strong evidence for clinical treatment selection from the perspective of metabolism. Metabolomics has been widely applied to quantify the changes in metabolites in cells, tissues, and entire organisms with the aim of studying the dynamic changes in endogenous metabolites and reflecting metabolic pathways and shifts in biological processes (Johnson et al., 2016). Therefore, this study aimed to clarify the metabolic changes in patients’ serum after treatment with EGFR-TKIs and PD-1/PD-L1 inhibitors and identify metabolic reprogramming mechanisms, thus providing new evidence for targeted therapy and antitumor immunotherapy in NSCLC.

## 2 Materials and methods

### 2.1 Study design and participants

Between November 2020 and November 2021, 120 patients with pathologically diagnosed NSCLC were enrolled in this study: 35 patients had not yet received preoperative surgery, radiotherapy, or chemotherapy (A group), 47 patients were treated with EGFR-TKIs (B group), and 50 patients were treated with PD-1/PD-L1 inhibitors (C group). Healthy volunteers without a known chronic or major disease and who were not undergoing any treatment were matched for age, sex, and smoking status with the enrolled patients (HC group). Patients with symptoms associated with bacterial infection, such as fever, increased leukocyte and neutrophil counts, and inflammation indicated by lung imaging or microculture, were excluded from the study to avoid any influence of bacterial infection on the serum metabolome.

### 2.2 Sample collection and preparation for metabolomics

Blood samples in B and C groups were collected after 2–3 treatment cycles with EGFR-TKIs or PD-1/PD-L1 inhibitors. Briefly, all serum samples were collected in the morning after an overnight fast. Whole blood (5 ml) was collected in sterile coagulation BD vacuum blood collection tubes. The tubes were gently shaken and centrifuged at 3,000 rpm for 10 min at room temperature. The supernatant (serum) was collected in 1.5-ml microfuge tubes and stored at −80°C until further analyses.

After thawing on ice, small metabolites from a 100-μlaliquot of serum was extracted by vortex-mixing with 300 μl of a methanol:acetonitrile (1:1 v/v) solution. The mixture was vortexed, sonicated, and then incubated at −20°C for 30 min. After centrifugation at 13,000 ×g at 4°C for 15 min, the supernatants were filtered through a 0.22-µm microporous membrane and carefully transferred to a sample bottle for LC-MS/MS analysis. Aliquots of all serum samples (10 µl) were pooled as part of the system adjustment and quality control (QC) process to prepare QC samples. The QC samples were treated in the same manner as the analytical samples.

### 2.3 Metabolite detection

Metabolomics analysis was perfomed on an ultra-performance liquid chromatography (UPLC) system (Agilent1290 Infinity II; Agilent Technologies Inc., CA, United States) connecting to a high-resolution tandem mass spectrometer (TripleTOF 5,600 Plus; AB SCIEX, Framingham, MA, United States). An ACQUITY HSS T3 column (100 × 2.1 mm, i. d. 1.8 µm; Waters, Milford, United States) were equipped for reversed-phase separation. The mobile phase consisted of solvent A (water, 0.1% formic acid) and solvent B (acetonitrile, 0.1% formic acid) with a gradient as previously described (Xu et al., 2021). The column temperature was maintained at 30°C and the flow rate was 0.30 ml/min.

For MS analysis, data acquisition was performed in full scan mode combined with independent data acquisition (IDA)-based auto-MS2 mode. Parameters of mass spectrometer were set as follows: m/z range: 80–1,000 (+) and 80–1,000 (−), ionspray voltage floating: 5500 V (+) and -4500 V (-), declustering potential: 80 V (+) and -80 V (−), collision energy: 10 V (+) and -10 V (-), interface heater temperature: 550^o^C (+) and 550^o^C (−), curtain gas: 35 psi, ion source gas 1 and ion source gas 2: 55 psi (+) and 55 psi (−). The m/z range of IDA analysis was set at 50–1,000 in both positive ion mode and negative mode, the collision energy was 35 V in positive ion mode and −35 V in negative ion mode, and collision energy spread was 15 V in both positive and negative ion modes.

During the entire period, the mass accuracy was calibrated after every six samples. Additionally, the purpose of introducing QCs every 10 samples in analytical sequence is to evaluate the reliability of large-scale metabolomics analysis.

### 2.4 Metabolomics analysis and annotation

Analysis of raw data obtained from UPLC-TOF/MS was conducted using the qualitative analysis software Analyst TF (version 1.7.1, AB SCIEX) for peak identification and comparison. As part of the metabolomics data processing workflow, peak picking, quality assessment, missing value imputation, normalisation, transformation, and scaling were performed. The details was present as below: 1) XCMS algorithm is applied to extract peaks using One-Map/PTO software (www.5omics.com) developed by Dalian ChemDataSolution Information Technology Co. Ltd. 2) Data quality is analyzed based on QC samples’ stability after peak extraction. The proportions of RSDs of mass spectrometry characteristics below 50% should account for more than 80%. QC calibration is performed using the MetNormalizer method based on support vector regression analysis. 3) The 80/20 rule is employed to eliminate metabolic features with non-zero values exceeding 20% in any category. Missing values are filled with the minimum value in the data. 4) A normalization process is required for that concentration of metabolites varies between individual organisms or during sample collection. To eliminate or reduce this heterogeneity, each metabolite is divided by the total concentration of the sample, so as to correct the influence of individual differences or other factors on the absolute concentration of metabolites. 5) In the data analysis, auto scaling and pareto scaling are used (scaling is used to eliminate variation in metabolite concentration orders of magnitude). Auto scaling is performed on the characteristic variables during principal component analysis (PCA) and partial least squares-discriminant analysis (PLS-DA), while pareto scaling is performed on the characteristic variables during orthogonal partial least squares-discriminant analysis (OPLS-DA).

The standard database (containing information of 1,550 metabolic standards), and custom databases including METLIN (http://metlin.scripps.edu/), Kyoto Encyclopedia of Genes and Genomes (KEGG) (http://www.kegg.jp/kegg/pathway.html), LipidMaps (https://www.lipidmaps.org/), Human Metabolome DataBase (HMDB) (https://hmdb.ca/), MassBank (https://massbank.eu/), and PubChem Database (https://pubchem.ncbi.nlm.nih.gov/) were used to validate, match, and annotate the processed molecular weights of the metabolites for accurate metabolite characterization. Notably, according to the definitions of metabolite identification as described by Schrimpe-Rutledge (Schrimpe-Rutledge et al., 2016), all the metabolites determined here would be considered as putative identification (Level 2), which lack the reference standard acquisition but used MS/MS data in combination of precursor m/z and retention time to derive the structural information.

SIMCA 15.0.2 (Umetrics AB, Umea, Sweden) was used to perform multivariate analyses. An unsupervised, nontargeted PCA was conducted using LC-MS/MS data, allowing for the visualization of holistic metabolome variation among groups and monitoring of stability over time. Significantly different metabolites were identified using OPLS-DA. Model parameters R2 and Q2 were used to assess model validity and avoid overfitting by supplying information about interpretability and predictability. OPLS-DA was applied to compute variable importance in projection (VIP). In a single-dimension statistical analysis, *p*-values were estimated through paired Student’s t-tests. The *t*-test, in conjunction with the OPLS-DA method, was used to determine the difference in metabolites between groups (while fulfilling VIP >1 and *p* < 0.05).

### 2.5 Statistical analysis

Statistics were conducted utilizing SPSS 25.0 (International Business Machines Corp., Armonk, NY, United States). Means and standard deviations of normally distributed data were calculated and reported. ANOVA was used for comparisons among multiple groups in the case of homogeneity of variance, followed by LSD *t*-test; otherwise, Welch’s *t*-test and Dunnett’s T3 test were adopted. A median (interquartile range) [M (P25, P75)] is calculated for non-normally distributed data. Comparing differences between groups was performed using the independent-sample Kruskal–Wallis test. A chi-square test was used to compare count data among groups Statistical significance was set at *p* < 0.05.

## 3 Results

### 3.1 Population and clinical characteristics

Characteristics of the study population are presented in Table 1. Forty-seven patients (group B) treated with EGFR-TKIs were analyzed, 29 of whom were treated with EGFR-TKIs as first-line therapy and 18 of whom were treated with EGFR-TKIs as second- and third-line therapy after cytotoxic chemotherapy. Twenty-four enrolled patients received PD-1/PD-L1 inhibitors alone as first-line treatment when their tumors had a PD-L1 expression greater than or equal to 50%, and 26 patients received PD-1/PD-L1 inhibitors as second- or third-line treatment after platinum failure regardless of the PD-L1 expression value. No significant differences in sex or age were identified among the patient groups. There were more men than women. Weight loss was observed in the EGFR-TKI and PD-1/PD-L1 inhibitor groups (B and C groups). Compared to the HC group, NSCLC patients had higher levels of CEA and NSE, whereas there was no difference in proGRP among the groups.

**TABLE 1:** The clinical characteristics of the enroled participants.

Characteristics	A (n = 35)	B (n = 47)	C (n = 50)	HC (n = 50)	χ2, P
Male/Female	25/10	30/17	40/10	38/12	5.370, 0.157
Age (years)	57.00 (52.00, 63.00)	57.00 (52.00, 66.00)	58.00 (53.00, 61.00)	54.50 (50.75, 58.00)	7.572, 0.056
Weight (Kg)	65.00 (60.00, 72.50)	61.00 (56.00, 67.00)^a^	60.00 (54.00, 67.00)^a^	67.00 (62.00, 70.00)^b,c^	17.979, <0.001
Type (n)	—	—	—	—	—
*Squamous carcinoma*	8	4	8	—	—
*Adenocarinoma*	17	27	34	—	—
*Large cell carcinoma*	2	2	1	—	—
*NA*	8	14	7	—	—
Tumor stage (n)	—	—	—	—	—
*Ⅰ*	3	3	2	—	—
*Ⅱ*	3	2	4	—	—
*Ⅲ*	5	6	9	—	—
*Ⅳ*	14	23	26	—	—
*NA*	10	13	9	—	—
Metastasis (n)	19	31	39	—	—
CEA (ng/ml)	3.76 (1.27, 27.82)	3.66 (2.36, 26.82)	3.92 (1.27, 10.64)	1.25 (1.00, 1.87)^a,b,c^	20.250, <0.001
NSE (μg/L)	7.67 (5.65, 9.78)	8.62 (6.32, 10.90)	7.56 (5.41, 9.95)	5.94 (5.32, 7.67)^a,b,c^	21.682, <0.001
proGRP (ng/ml)	0.04 (0.03, 0.06)	0.06 (0.03, 0.205)	0.04 (0.03, 0.07)	0.05 (0.04, 0.06)	5.385, 0.146

Note: A, NSCLC, patients without any anticancer treatment; B, NSCLC, patients treated with EGFR-TKIs; C, NSCLC, patients treated with PD-1/PD-L1, inhibitors; HC, healthy control. ^a^Compared with the A group, *p* < 0.05; ^b^Compared with the B group, *p* < 0.05;^c^Compared with the C group, *p* < 0.05.

### 3.2 Multivariate statistical analysis of metabolites

Liquid chromatography-mass spectrometry is a method that is commonly used to analyze metabolomic data and generate the mass-to-charge ratio for metabolomic analysis of biological samples. Processed data comprising retention time, exact mass, and peak intensity from the three subgroups, including the untreated group (group A), the EGFR-TKI-treated group (group B), and the PD-1/PD-L1 inhibitor-treated group (group C), were subjected to multivariate statistical analysis.

**FIGURE 1:**
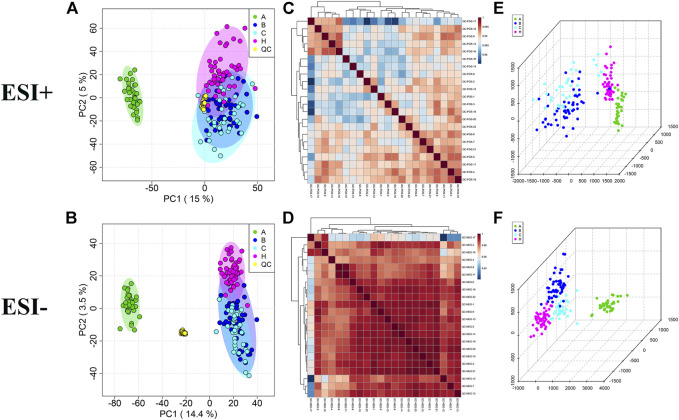
Multivariate statistical analysis in A, B, C and HC groups. **(A,B)** PCA score plots with QC samples; **(C,D)** Correlation analysis of QC samples in the ESI+ and ESI− scan modes. **(E,F)** OPLS-DA score plots of A, B, C and HC groups in the ESI+ and ESI− scan modes.

PCA plots showed a clustering of QC samples (Figures 1A,B), and correlation heatmaps present strong correlations between QC samples (Figures 1C,D), indicating the satisfactory stability and repeatability of the analytical system. However, no distinct classifications were achieved for the A vs B, A vs C, or B vs C comparisons in either the positive or negative ion modes, indicating no effective separation of the principal components.

**FIGURE 2:**
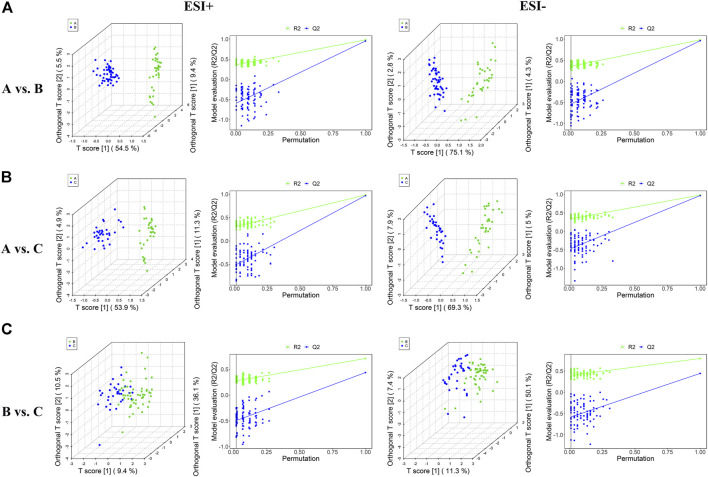
Plots of OPLS-DA score and permutation testing for A vs. B **(A)**, A vs. C **(B)**, B vs. C **(C)** comparisons in the ESI+ and ESI− scan modes. The criterion for evaluating whether there is overfitting in the OPLS-DA model is that the regression line at a blue Q2 point crosses or is less than 0 from the abscissa.

As OPLS-DA is more discriminatory than PCA, it was further used to explore the different metabolic profiles. Distinct clustering in the A vs B, A vs C, and B vs C comparisons was observed in both the positive and negative ion modes, indicating a clear separation of the three patient cohorts (Figures 1E,F and Figure 2). Model evaluation used OPLS-DA’s R2X, R2Y, and Q2 (cumulative) parameters. Table 2 list these modeling parameters within each comparison. The high Q2 values of the OPLS-DA model showed its high accuracy. Overfitting of the supervised OPLS-DA models was examined by performing 200 random permutations. Positive and negative ions had Q2 distributions with Y-intercepts lower than zero, indicating the reliability of OPLS-DA (Figure 2). Therefore, the PCA and OPLS-DA models showed significant distinctions among the A vs B, A vs C, and B vs C comparisons and were highly effective in characterizing serum metabolites.

**TABLE 2:** Comparisons among groups under ESI+ and ESI- scan modes using PLS-DA and OPLS-DA analysis models.

Scan mode	Analysis model	Group	R2X	R2Y	Q2
ESI+	PLS-DA	4 group	0.27	0.95	0.95
	OPLS-DA	4 group	0.33	0.65	0.57
		A vs. B	0.55	0.90	0.90
		A vs. C	0.54	0.89	0.88
		B vs. C	0.36	0.42	0.31
ESI-	PLS-DA	4 group	0.23	0.99	0.99
	OPLS-DA	4 group	0.38	0.77	0.574
		A vs. B	0.75	0.96	0.96
		A vs. C	0.69	0.96	0.96
		B vs. C	0.50	0.59	0.52

Note: A, NSCLC, patients without any anticancer treatment; B, NSCLC, patients treated with EGFR-TKIs; C, NSCLC, patients treated with PD-1/PD-L1, inhibitors; HC, healthy control.

### 3.3 Differential metabolite analysis and identification

**FIGURE 3:**
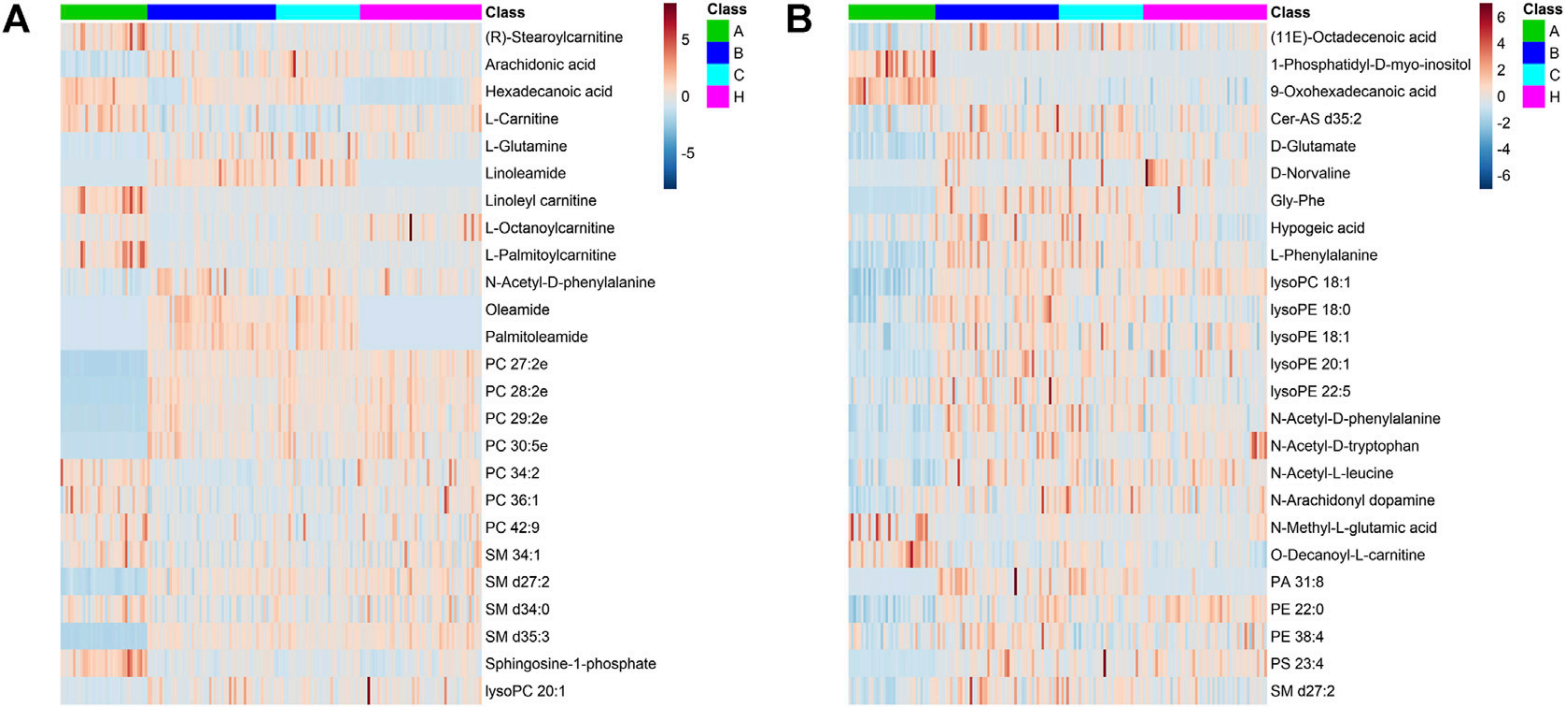
Differential metabolite heat maps in ESI+ **(A)** and ESI− **(B)** scan modes. The columns represent samples, the rows represent metabolites, and the relative content of the metabolites is displayed by color. The heat map shows differential metabolites among A, B, C, and HC groups.

Databases, publically available and integrated, were used for qualitative identification. Using the positive and negative ion modes, 754 and 697 metabolites were identified, respectively. Subsequently, 97 and 87 different metabolites in the A vs B and A vs C comparisons were selected using a fold-change threshold >1.5 or <2/3, VIP >1, and Student’s t-test threshold *p* < 0.05. Thirty-four different metabolites in the B vs C comparison were selected using a fold-change threshold >1.2 or <2/3, VIP >1, and Student’s t-test threshold *p* < 0.05. There was clear clustering in heat maps of 25 representative differential metabolites detected in both positive and negative modes between groups A, B, C, and HC (Figure 3), consistent with the OPLS-DA results.

**FIGURE 4:**
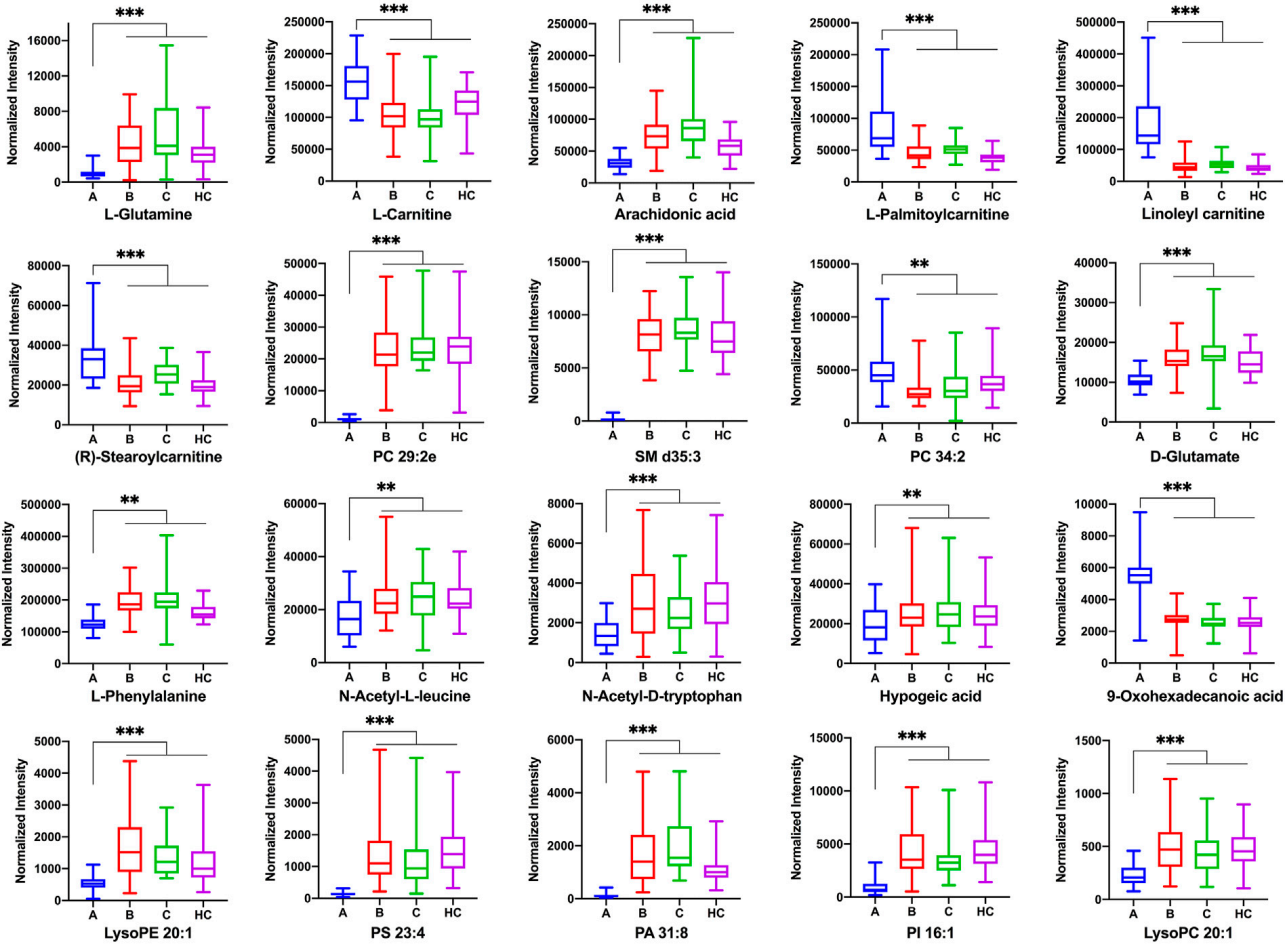
The normalized peak intensity of 20 representative differential metabolites among A, B, C, and HC groups. p** < 0.01; p*** < 0.001.

The most abundant classes of metabolites for the A vs B and A vs C comparisons were carnitines, amino acids, fatty acids, and lipids (Table 3), whereas for the B vs C comparison, the most abundant classes were fatty acids and lipids (Table 4). In the A vs B and A vs C comparisons, the levels of most carnitines decreased, whereas the levels of essential amino acids increased. Fatty acids and lipids were both increased and decreased because of their wide variety. Twenty significantly altered metabolites were identified based on the screening criteria (FC > 1.5 or <2/3, VIP >1, and *p* < 0.05). Semi-quantitative analysis of these representative differential metabolites showed that after treatment with EGFR-TKIs or PD-1/PD-L1 inhibitors, the levels of the metabolites were closer to those in the HC group (Figure 4), which proved the effectiveness of the treatment. Notably, some glycerophosphatide (mainly PC and PA) levels in the treatment groups did not return to normal, and an increase of arachidonic acid in B and C groups was still observed as compared with healthy individuals (data not shown).

**TABLE 3:** List of statistically significant metabolites in A vs B and A vs C comparisons.

Metabolites		B vs. A				C vs. A			
		VIP	Log2(FC)	P	Trend	VIP	Log2(FC)	P	Trend
Carnitine	l-Carnitine	1.77	-0.70	<0.001	↓	1.94	-0.89	<0.001	↓
	(R)-Stearoylcarnitine	1.55	-0.76	<0.001	↓	1.25	-0.59	<0.001	↓
	l-Palmitoylcarnitine	1.57	-1.11	<0.001	↓	1.35	-1.01	<0.001	↓
	Linoleyl carnitine	2.01	-2.05	<0.001	↓	1.80	-1.96	<0.001	↓
Amino acids	l-Glutamine	1.65	2.02	<0.001	↑	1.61	2.28	<0.001	↑
	D-Glutamate	2.00	0.81	<0.001	↑	1.90	0.83	<0.001	↑
	l-Phenylalanine	2.05	0.85	<0.001	↑	1.90	0.81	<0.001	↑
	N-Acetyl-l-leucine	1.26	0.69	<0.001	↑	1.14	0.59	<0.001	↑
	N-Acetyl-d-tryptophan	1.41	1.39	<0.001	↑	1.38	1.01	<0.001	↑
Fatty acids	Arachidonic acid	1.82	1.67	<0.001	↑	1.99	1.73	<0.001	↑
	Hypogeic acid	1.09	0.66	<0.001	↑	1.18	0.67	<0.001	↑
	9-Oxohexadecanoic acid	1.99	-0.77	<0.001	↓	2.00	-0.96	<0.001	↓
Lipids	PI 16:1	1.67	2.21	<0.001	↑	1.81	1.91	<0.001	↑
	Lyso PE 20:1	1.76	1.80	<0.001	↑	1.85	1.50	<0.001	↑
	Lyso PC 20:1	1.60	1.37	<0.001	↑	1.73	1.14	<0.001	↑
	PA 31:8	1.48	4.16	<0.001	↑	2.02	4.13	<0.001	↑
	PS 23:4	1.65	3.54	<0.001	↑	1.28	3.35	<0.001	↑
	PC 34:2	1.67	-0.82	<0.001	↓	1.17	-0.66	<0.001	↓
	PC 29:2e	2.36	4.25	<0.001	↑	2.38	4.20	<0.001	↑
	SM d35:3	2.53	5.46	<0.001	↑	2.42	5.45	<0.001	↑

Note: A, NSCLC, patients without any anticancer treatment; B, NSCLC, patients treated with EGFR-TKIs; C, NSCLC, patients treated with PD-1/PD-L1, inhibitors; HC, healthy control. VIP, variable influence on projection; FC, fold-change.

**TABLE 4:** List of statistically significant metabolites in B vs C comparison.

Metabolites		B vs. C			
		VIP	Log2(FC)	P	Trend
Amino acids	N-Acetyl-d-tryptophan	1.15	0.38	0.044	↑
	l-Kynurenine	1.57	-0.27	0.027	↓
	l-Isoleucine	1.26	0.96	0.045	↑
	Arachidonoyl Glycine-d8	1.45	-0.43	0.044	↓
Lipids	PI 38:3	1.38	0.35	0.018	↑
	PI 38:5	1.47	0.32	0.012	↑
	PE 38:4	2.54	0.64	<0.001	↑
	PC 38:4	1.53	0.37	0.020	↑
	LysoPE 18:0	2.38	0.33	<0.001	↑
	LysoPC 18:3	2.19	0.49	<0.001	↑

Note: A, NSCLC, patients without any anticancer treatment; B, NSCLC, patients treated with EGFR-TKIs; C, NSCLC, patients treated with PD-1/PD-L1, inhibitors; HC, healthy control. VIP, variable influence on projection; FC, fold-change.

Ten significantly altered metabolites were identified in the B vs C comparison based on FC > 1.2 or <2/3, VIP >1, and *p* < 0.05. The main differentially regulated metabolites between the two groups were amino acids and lipids. Among them, tryptophan and its metabolite kynurenine, phosphatidylinositol, phosphatidylethanolamine, phosphatidylcholine, lysophosphatidylethanolamine, and lysophosphatidylcholine changed significantly.

### 3.4 Perturbed pathways identified in group comparisons

**FIGURE 5:**
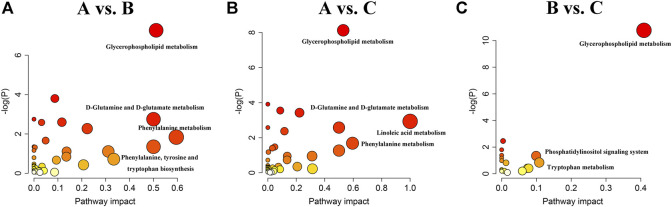
Summary of metabolic pathways analyzed in A vs. B **(A)**, A vs. C **(B)** and B vs. C **(C)** comparisons.

We next examined the metabolic pathways enriched among the differential metabolites. Pathway impact values refer to the cumulative percentage from the matched metabolite nodes and the maximum importance of each pathway is 1. The results of metabolic pathway analysis showed that the perturbed pathways were mainly enriched in 1) phenylalanine metabolism, glycerophospholipid metabolism, D-glutamine and D-glutamate metabolism, and phenylalanine, tyrosine, and tryptophan biosynthesis for the A vs B comparison; 2) linoleic acid metabolism, phenylalanine metabolism, glycerophospholipid metabolism, and D-glutamine and D-glutamate metabolism for the A vs C comparison; and 3) glycerophospholipid metabolism, tryptophan metabolism, and phosphatidylinositol signaling system for the B vs C comparison (Figure 5 and Table 5).

**TABLE 5:** Significantly altered metabolic pathways in A vs B, A vs C and B vs C comparisons.

Comparison	Pathway name	KEGG.id	-log(P)	Impact	Hits
A vs B	Phenylalanine metabolism	hsa00360	1.84	0.60	2
	Glycerophospholipid metabolism	hsa00564	7.27	0.51	9
	D-Glutamine and D-glutamate metabolism	hsa00471	2.75	0.50	2
	Phenylalanine, tyrosine and tryptophan biosynthesis	hsa00400	1.35	0.50	1
A vs C	Linoleic acid metabolism	hsa00591	2.93	1	2
	Phenylalanine metabolism	hsa00360	1.67	0.59	2
	Glycerophospholipid metabolism	hsa00564	8.12	0.53	10
	D-Glutamine and D-glutamate metabolism	hsa00471	2.58	0.50	2
B vs C	Glycerophospholipid metabolism	hsa00564	10.77	0.41	8
	Tryptophan metabolism	hsa00380	0.84	0.11	2
	Phosphatidylinositol signaling system	hsa04070	1.34	0.10	2

Note: A, NSCLC, patients without any anticancer treatment; B, NSCLC, patients treated with EGFR-TKIs; C, NSCLC, patients treated with PD-1/PD-L1, inhibitors; HC, healthy control.

There were three shared metabolic pathways in the A vs B and A vs C comparisons, all of which were related to lipid and amino acid metabolism. The two groups shared many of the same differential metabolites and thus shared the same enriched metabolic pathways. Although both EGFR-TKIs and PD-1/PD-L1 inhibitors can regulate tumor lipid metabolism, our results suggest that the mechanisms of lipid metabolism regulation may differ, leading to significant differences in lipid metabolism between the two treatments. As expected, the enriched metabolic pathways in the B vs C comparison included the tryptophan metabolic pathway. Moreover, glycerophospholipid metabolism was the most significantly altered metabolic pathway in all three comparisons, suggesting that lipid metabolism plays an important role in tumor development.

## 4 Discussion

The incidence and mortality of lung cancer, especially NSCLC, are very high. Although there are many treatments for NSCLC, the underlying mechanisms of the treatment effects remain unclear. In this study, we used a nontargeted metabolomics approach based on liquid chromatography-mass spectrometry to analyze the metabolic response of NSCLC patients to EGFR-TKIs or PD-1/PD-L1 inhibitors in an attempt to provide new clues to identifying the antitumor mechanism of these two treatments from the perspective of metabolism.

Many studies have shown that EGFR-TKIs and PD-1/PD-L1 inhibitors can affect tumor metabolism (Chang et al., 2015; Zhang et al., 2021). EGFR-TKIs inhibit tumor growth by blocking the activation of EGFR in cancer cells and the downstream MAPK (RAS/RAF/MEK/ERK) and PI3K/AKT/mTOR signaling pathways. They inhibit cell proliferation and tumor-induced angiogenesis while promoting apoptosis (Singh et al., 2021). It has been reported that the overactivation of the MAPK and PI3K/AKT pathways is related to the reprogramming of specific metabolic processes, including increasing glucose uptake through glucose transporter 1, enhancing glutamine replenishment by activating glutamate pyruvate aminotransferase 2, and cellular lipid reprogramming (Koundouros and Poulogiannis, 2020). Besides, PD-1/PD-L1 inhibitors were originally designed to reactivate the host antitumor immune response by blocking the PD-1/PD-L1 immune checkpoint. However, there is increasing evidence that immune checkpoint inhibitors can affect the metabolic fitness of tumor and T cells. For example, the expression of PD-L1 and B7-H3 (also known as CD276) in tumor cells can stimulate aerobic glycolysis in tumor cells by activating the PI3K/AKT/mTOR pathway (Li et al., 2019a; Stirling et al., 2022). Conversely, the interaction of PD-1 with PD-L1 or PD-L2 can impair the metabolic reprogramming of T cells by inhibiting the similar pathway (Wang et al., 2022). Therefore, these studies provide theoretical support for us to explore the mechanisms of these two emerging treatment methods for NSCLC from the perspective of metabolism. To the best of our knowledge, there has been no metabolomic study in NSCLC patients for comparing the EGFR-TKIs and PD-1/PD-L1 inhibitors.

Our PCA score chart showed that under the positive and negative ion modes, the clusters of the EGFR-TKI- and PD-1/PD-L1 inhibitor-treated groups tended to be close to the cluster of the HC group and were significantly separated from the cluster of the primary lung cancer group, indicating that EGFR-TKIs and PD-1/PD-L1 inhibitors have definite antitumor therapeutic effects on NSCLC. The OPLS-DA score charts showed good differentiation between the groups. Compared with the newly diagnosed, untreated lung cancer group, there were significant changes in carnitine, amino acids, fatty acids, and lipids in the EGFR-TKI- and PD-1/PD-L1 inhibitor-treated groups. The treatment groups had two similar significantly altered metabolic pathways: glutamine and D-glutamate metabolism and glycerol phospholipid metabolism. We believe that this may be related to the action of therapeutic drugs on the same signaling pathway (such as PI3K/AKT/mTOR) and the utilization of the same therapeutic drugs (such as platinum) in the early treatment stage. After treatment with EGFR-TKIs or PD-1/PD-L1 inhibitors, glutamate/glutamine, phenylalanine, n-acetyl-l-leucine, n-acetyl-d-tryptophan, D-n-valine, arachidonic acid, and linoleic acid levels were significantly increased in patients with NSCLC, whereas carnitine, stearyl carnitine, palmitoyl carnitine, linoleic carnitine, and palmitic acid levels were markedly decreased. The levels of these metabolites in the two treatment groups were similar to those in the HC group, which is consistent with the results of the PCA.

Lung cancer cells require glutamine to meet their metabolic needs. As a nitrogen source, glutamine directly or indirectly after conversion to glutamate contributes to many anabolic processes in cancer, such as the biosynthesis of amino acids, nucleotide bases, and hexosamine. It also plays an important role in redox homeostasis. In addition, in a process known as glutamine decomposition, glutamine is converted to α-ketoglutaric acid, which serves as the energy and carbon source that supplements intermediates of the tricarboxylic acid cycle (Tang et al., 2022; Vanhove et al., 2019). EGFR phosphorylates ELK1 through the MEK/ERK pathway, which can activate GDH1 transcription and glutamine degradation, providing a new perspective for changes in glutamine metabolism in tumor cells (Yang et al., 2020). In addition, as mentioned earlier, the interaction between PD-1 and PD-L1 or PD-L2 inhibits the PI3K/AKT/mTOR pathway and blocks metabolic reprogramming of T cells, including glutamine hydrolysis. In our study, EGFR-TKIs and PD-1/PD-L1 inhibitors reduced glutamine decomposition, which may be related to the inhibition of the MEK/ERK and/or PI3K/AKT/mTOR pathways. Further, the level of glutamine increased significantly in our treatment groups, indicating a reduced consumption of glutamine after treatment, which may also reflect the reduction in glutamine addiction in tumor cells after treatment (Euceda et al., 2017).

Essential amino acids (tryptophan, methionine, valine, lysine, isoleucine, phenylalanine, leucine, threonine, and histidine) not only provide raw materials for the synthesis of biological macromolecules such as proteins, lipids, and nucleic acids but can also be used as signaling molecules to induce the activation of the mTOR pathway (Hosios et al., 2016). Driven by RAS/RAF/MEK/ERK and PI3K/AKT/mTOR signaling, proliferating cells import nutrients, such as amino acids, thus activating mTORC1, inducing transcriptional reprogramming of MYC and other transcription factors, and promoting the expression of growth signal-related genes and protein and ribosome synthesis (Stine et al., 2022). We speculate that EGFR-TKIs and PD-1/PD-L1 inhibitors block PI3K/AKT/mTOR signaling, which blocks the absorption of essential amino acids by tumor cells, resulting in increased phenylalanine, n-acetyl-l-leucine, and n-acetyl-d-tryptophan. In addition, the increase in phenylalanine may be closely related to cell cycle arrest in the G1 phase. Previous studies have shown that the EGFR-TKIs gefitinib and lapatinib can block cell cycle progression (Konecny et al., 2006; Rusnak et al., 2001). This also demonstrates the effectiveness of EGFR-TKIs in EGFR-mutated NSCLC.

Arachidonic acid, linoleic acid, and palmitic acid are involved in fatty acid metabolism. Fatty acids can participate in the structural synthesis of phospholipids on the membrane of cancer cells and promote the transduction of important signals. Cancer cells also utilize ATP produced by fatty acid β-oxidation as an energy source (Koundouros and Poulogiannis, 2020). Carnitine is an essential energy substance involved in the β-oxidation of fatty acids (Longo et al., 2016); the rate-limiting step of β-oxidation is the carnitine shuttle (Li and Zhao, 2021). Our study found that carnitine levels were decreased after treatment with EGFR-TKIs or PD-1/PD-L1 inhibitors. This may inhibit the β-oxidation of fatty acids, leading to the abnormal metabolism of fatty acids (arachidonic acid, linoleic acid, and palmitic acid) and reducing the energy uptake of tumor cells. These results suggest that EGFR-TKI or PD-1/PD-L1 inhibitor treatment may improve the effect of antitumor therapy by restoring endogenous fatty acid homeostasis. The significantly differentially regulated metabolites in these patients, including arachidonic acid, linoleic acid, and glutamate, are all involved in inflammation and oxidation. It has been suggested that EGFR-TKIs or PD-1/PD-L1 inhibitors may also play a therapeutic role in NSCLC through anti-inflammatory and antioxidant mechanisms (Liao et al., 2012).

Metabolomic profiling of tumor and plasma samples from NSCLC patients has indicated alterations in the lipid composition. Lipids are used as energy sources and cellular components (in the form of phospholipids) for rapidly proliferating cancer cells (Kowalczyk et al., 2021). Glycerophospholipid metabolism is highly related to the development and progression of cancer (Yang et al., 2022). A review of 12 articles showed that the phosphatidylcholine, phosphatidylethanolamine, phosphatidylinositol, cardiolipin, phosphatidylserine, phosphatidylglycerol, ceramide, lysophosphatidylethanolamine, lysophosphatidylcholine, and lysophosphatidylglycerol levels were significantly different between NSCLC and normal tissues (Jianyong et al., 2021), which was consistent with what we observed in the serum of NSCLC patients. After treatment with EGFR-TKIs or PD-1/PD-L1 inhibitors, the levels of various glycerol phospholipids tended to decrease to levels similar to those in the HC group, indicating that these two treatments may block the dysregulation of lipid metabolism in tumor cells to a certain extent to exert antitumor therapeutic effects. In addition, changes in lipid composition can alter the properties of the cell membrane and affect its function, including material exchange and signal transduction (Lin et al., 2017). The interference of EGFR-TKIs or PD-1/PD-L1 inhibitors on the metabolism of glycerophospholipids or glycerol esters in patients with NSCLC suggests that small-molecule targeted drugs or immunotherapies may also inhibit the malignant proliferation of tumor cells by interfering with cell membrane stability. Our results also suggest that lipid differentials may be good biomarkers for tumorigenesis, development, and prognosis.

There were significant differences in lipid metabolism (glycerophospholipid metabolism and phosphatidylinositol signaling) and amino acid metabolism (tryptophan metabolism) between the EGFR-TKI and PD-1/PD-L1 inhibitor groups. The therapeutic mechanisms of the two treatments are not identical, which leads to differences in the levels and downstream effectors of various glycerophospholipids. We also found that PD-1/PD-L1 inhibitor treatment affected phosphatidylinositol signaling more than EGFR-TKI treatment. Phosphatidylinositol is a lipid signaling molecule that is the main regulator of cell signaling (Hammond and Burke, 2020). When PD-1 binds to PD-L1, downstream T cell activation is blocked by the phosphorylation of phosphatidylinositol-3-kinase. Conversely, blocking the PD-1/PD-L1 signaling pathway can inhibit the phosphatidylinositol-3-kinase/AKT signaling pathway and promote T cell activation. Therefore, phosphatidylinositol signaling may be more related to PD-1/PD-L1 inhibitor therapy. Futhermore, the conversion of tryptophan to kynurenine can also significantly affect the response to immunotherapy in cancer (Li et al., 2019b). Tryptophan in the tumor microenvironment can be metabolized to kynurenine by indoleamine-2,3-dioxygenase. Kynurenine inhibits T cell activation, which allows tumor cells to evade immune system monitoring and clearance. In our study, the level of kynurenine was slightly increased in the PD-1/PD-L1 inhibitor group. We speculate that this might be related to PD-1/PD-L1 inhibitor resistance in some patients with NSCLC (Lei et al., 2020). This suggests that inhibiting the tryptophan-kynurenine pathway through the administration of indoleamine-2,3-dioxygenase inhibitors may benefit NSCLC patients who are resistant to PD-1/PD-L1 inhibitors (Kotecki et al., 2021).

Compared with healthy controls, patients treated with EGFR-TKIs and PD-1/PD-L1 inhibitors had abnormal glycerophosphatide metabolism. Some glycerophosphatide (mainly PC and PA) levels in the treatment groups did not return to normal, so the metabolic pathway of glycerophosphatide still differed from healthy individuals. In addition, a different metabolism of arachidonic acid was observed. Arachidonic acid metabolism is involved in inflammation and lipid oxidation processes. Tumor cells maintain their proliferation by metabolizing arachidonic acid. The arachidonic acid metabolic chain may be inhibited by EGFR-TKIs or PD-1/PD-L1 inhibitors, making it unable to produce pro-inflammatory and pro-tumor substances (such as eicosanoids) through key enzymes in the arachidonic acid metabolic network, thus causing a raise of the arachidonic acid (Koundouros et al., 2020). Consequently, treatment and healthy groups exhibit different arachidonic acid metabolic pathways. In addition, we noted that there were no significant differences in amino acid-related metabolic pathways (such as alanine, aspartic acid and glutamate metabolic pathways) between the healthy group and the treatment groups, suggesting that therapeutic drugs may restore endogenous amino acid to normal levels by regulating amino acid metabolism in NSCLC patients.

Altogether, our results show that EGFR-TKIs and PD-1/PD-L1 inhibitors induce changes in carnitine, amino acids, fatty acids, and lipids and alter related metabolic pathways in NSCLC patients. Changes in endogenous metabolism are caused by drug action and may be related to the effects of antitumor therapy. However, our research has some limitations. First, the sample size was small, so a large, multicenter study is necessary to reduce sampling error. Second, some of the included patients had different degrees of concomitant drug use in the early treatment stage, which inevitably affected metabolic pathways to some extent. Third, the types of EGFR-TKIs and PD-1/PD-L1 inhibitors were not completely consistent, which also led to slightly inconsistent results and needs to be standardized in further studies.

## Data Availability

The raw data supporting the conclusions of this article will be made available by the authors, without undue reservation.

## Abbreviations

EGFR: epidermal growth factor receptor; ERK: extracellular regulated protein kinase; HC: healthy control; MEK: mitogen-activated proteinkinase kinase; mTOR: mammalian target of rapamycin; NSCLC: non-small cell lung cancer; OPLS-DA: orthogonal partial least squares-discriminant analysis; PCA: principal component analysis; PD-1: Programmed death-1; PD-L1: Programmed death ligand 1; PI3K: Phosphoinositide 3-kinases; QC: quality control; TKI: tyrosine kinase inhibitor; VIP: variable importance in projection.
